# Yellow Fever

**Published:** 1878-10-20

**Authors:** 


### YELLOW FEVER.

The paper on the above topic, in the present issue of the RECORD, by Dr. Greene, of Macon, Ga., presents new and interesting points. It transcends the length usually allowed for our journal, but we give it a place by reason of the great interest in the sub'ect of yellow fever at the present time, and in the hope that the views of the writer may be realized as to the virtues of the Alum end Iron Mass in this awful disease. The fatality of the affection is certainly dreadful, and we think that any and everything promising any good in its treatment should be made known to the profession, and a test be made of its virtues. For like reasons we give place to Dr. Greenley's article.

